# Influence of seton configuration, thickness, and laxity on patient comfort in chronic perianal fistula: a prospective comparative study

**DOI:** 10.1007/s00384-025-04985-9

**Published:** 2025-08-25

**Authors:** J Barambio, Ana Leon-Bretscher, María Ramírez Bescos, Paula Soto García, Paloma Gadea Uria, Teresa Laloumet Garcimartin, Elena Viejo Martinez, Felipe Acedo Fernandez de Pedro, Patricia Ortega Domene, Alicia Ruiz de la Hermosa, María Luisa De Fuenmayor-Valera, Gloria Paseiro-Crespo

**Affiliations:** 1 Department of General and Digestive Surgery, Hospital Universitario Infanta Leonor, Avenida Gran Vía del Este 80, 28031 Madrid, Spain; 2 Universidad Complutense de Madrid, Madrid, Spain

**Keywords:** Perianal fistula, Seton drainage, O-shaped seton, V-shaped seton, Laxity, Seton thickness, Quality of life, Surgical techniques, Silicone vessel loop

## Abstract

**Purpose:**

To determine how seton configuration, thickness, and laxity influence patient comfort and clinical outcomes in the management of chronic perianal fistulas.

**Methods:**

A prospective single-center study was conducted involving 33 patients (53 setons). Both intraindividual paired (n = 20) and independent group analyses (n = 13) were performed. Setons were categorized by configuration (O-shaped vs. V-shaped), thickness (1.5 mm vs. 2.5 mm), and laxity (≤ 2 cm vs. > 2 cm from the anal verge). Patient-reported outcomes were assessed using structured questionnaires evaluating sitting discomfort, discharge, sexual impact, and fecal incontinence.

**Results:**

O-shaped setons caused significantly less sitting discomfort than V-shaped ones (50.0% vs. 80.0%, p = 0.021). Excess laxity (> 2 cm) was associated with increased discharge (86.4% vs. 51.6%, p = 0.008), sexual impact (42.1% vs. 10.3%, p = 0.016), and fecal incontinence (66.7% vs. 10.0%, p = 0.041). Thicker setons (2.5 mm) showed a non-significant trend toward more pain (42.9% vs. 17.9%, p = 0.080).

**Conclusion:**

Seton configuration and laxity significantly affect patient comfort. O-shaped setons and shorter laxity (≤ 2 cm) are preferable for reducing discomfort. These findings support evidence-based seton selection, although larger multicenter studies are needed to confirm these results.

**Supplementary Information:**

The online version contains supplementary material available at 10.1007/s00384-025-04985-9.

## Introduction

Chronic perianal fistula remains a clinical challenge, often requiring prolonged seton drainage. Despite widespread use, little evidence exists regarding how specific seton characteristics—such as configuration, thickness, or laxity—influence patient comfort and clinical outcomes [1–3].

Silicone Vessel Loop setons are commonly used due to their perceived comfort; however, technical decisions typically remain empirical and surgeon-dependent [4, 5]. To our knowledge, this represents the first prospective comparative evaluation of these specific technical aspects of seton placement.

This prospective comparative study aimed to evaluate the influence of seton configuration, thickness, and laxity on patient-reported comfort, providing clinically relevant insights to improve outcomes and patient satisfaction.

Although various materials are available for seton placement, we consistently used silicone vessel loops throughout the study. This material is widely adopted in clinical practice due to its flexibility, inertness, and ease of outpatient management. Its uniform use allowed us to ensure methodological consistency and to focus on the comparative assessment of technical features such as configuration, thickness, and laxity.

## Materials and methods

### Study design

This was a prospective, single-center, observational study conducted in the Department of General and Digestive Surgery of a secondary level hospital between December 2022 and December 2024. This manuscript adheres to the STROBE (Strengthening the Reporting of Observational Studies in Epidemiology) guidelines for reporting observational studies. The aim was to assess the influence of seton configuration, thickness, and laxity on clinical outcomes and patient-reported quality of life in cases of chronic perianal fistula.

Patients were included in one of two analysis groups based on their clinical course and follow-up duration. Some patients received only one seton configuration during treatment, while others underwent a configuration switch as part of their outpatient management. This allowed for both independent group comparisons and intraindividual analyses within the same patient. Group allocation was not randomized but reflected real-world clinical conditions in a pragmatic observational setting.

### Study population

Adults (≥ 18 years) with a diagnosis of chronic perianal fistula who required treatment with a silicone Vessel Loop seton were included. Patients with anal neoplasia, severe immunosuppression, or inability to complete follow-up were excluded. The final cohort comprised 33 patients (53 cases), some of whom received both seton configurations at different time points.

### Intervention

**Fig. 1:**
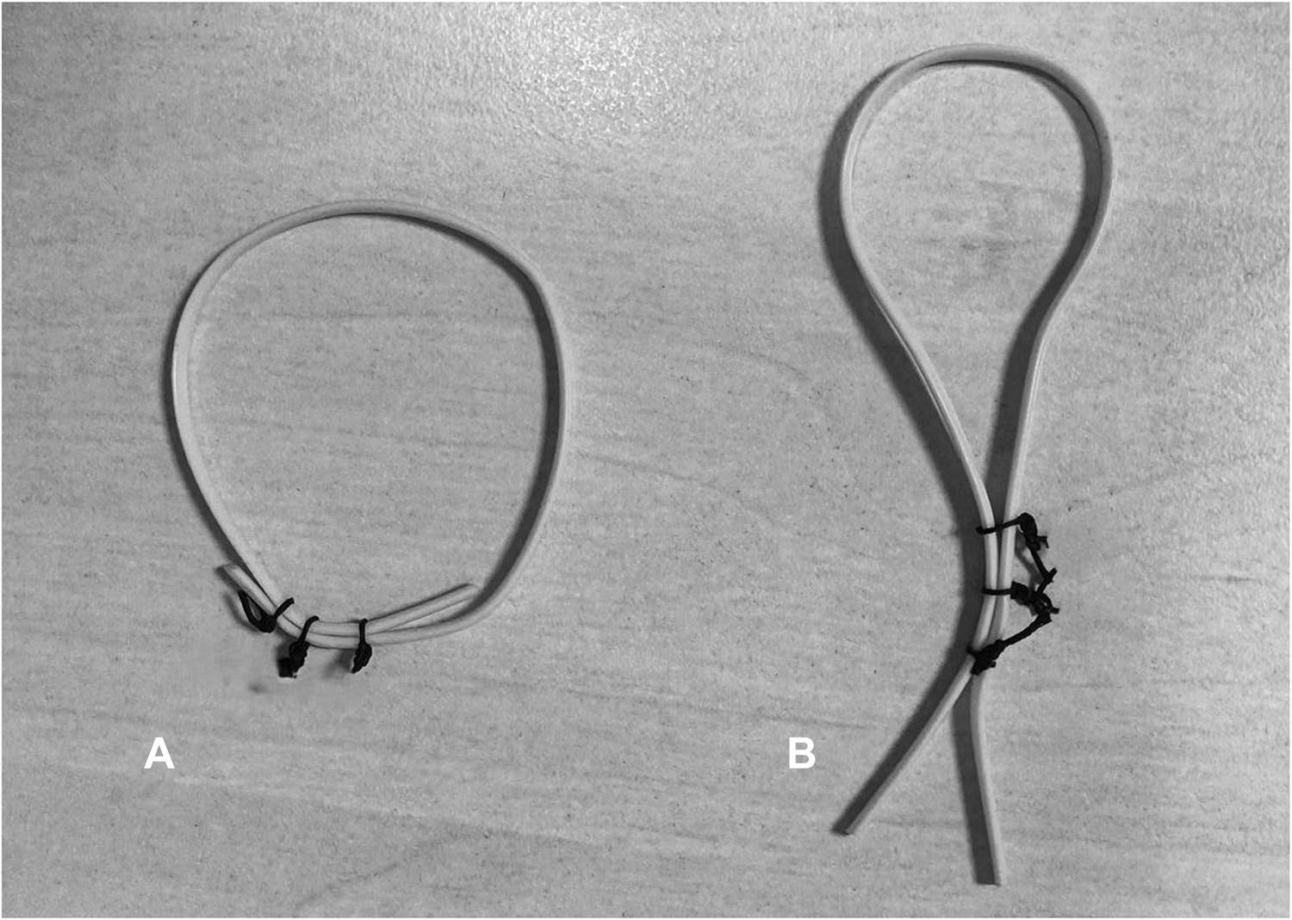
Schematic representation of the two seton configurations used in the study. (**A**). (**A**) O-shaped configuration, with the silicone loop tied upon itself, allowing rotational movement. (**B**) V-shaped configuration, with both ends aligned and directed externally, limiting mobility

All patients were treated with silicone Vessel Loop setons secured using three black 2–0 silk knots, cut flush at the knot level to facilitate perianal hygiene. Two placement techniques were used:

- O-shaped configuration: the seton was looped and tied upon itself, forming a closed circle around the fistulous tract, allowing greater rotational mobility.
- V-shaped configuration: the seton was tied without closing the loop, with both ends aligned and directed externally, limiting its mobility.
- Beyond configuration, setons were also categorized by:
- Thickness: thin (1.5 mm) or thick (2.5 mm), depending on Vessel Loop caliber.
- Laxity: categorized as ≤ 2 cm or > 2 cm (Fig. 1).

#### Measurement of seton laxity

Seton laxity was measured immediately after surgical placement, with patients in prone-jackknife position and buttocks laterally retracted using elastic tapes for optimal perianal exposure. A standardized sterile ruler was used, measuring from the skin surface to the apex of the seton at maximal extension without applying excessive tension (just before visible elastic stretching occurred). During outpatient replacements, laxity was similarly measured using the same ruler, with patients placed laterally in Sims'position.

Seton thickness and laxity were chosen based on surgeon preference at the time of placement, without predefined allocation criteria. These characteristics were subsequently evaluated in an exploratory, descriptive analysis of patient outcomes.

#### Outcome assessment

##### Intervention sequence

In the paired group (n = 20), patients received both seton configurations (O and V) in an alternating sequence, starting with an O-shaped seton in the first patient, followed by a V-shaped seton in the next, and so forth. This approach approximated a simple randomization procedure, aiming for a balanced distribution of configurations by the end of the study period. Each configuration was maintained for a minimum of three months.

In the unpaired group (n = 13), patients received only one seton configuration, which was selected by the surgeon at the time of placement based on clinical judgment, without predefined allocation criteria. The same minimum follow-up period of three months was applied in both groups.

No wash-out period was used between seton replacements in the paired group; configurations were switched directly during outpatient visits, without anesthesia or special preparation. No cases of seton loss occurred during these replacements.

#### Variables collected

Clinical, demographic, and patient-reported variables were prospectively collected for all cases. Demographic and clinical data included age, sex, body mass index (BMI), comorbidities (including obesity, hypertension, and diabetes mellitus), history of smoking, presence of Crohn’s disease, fistula characteristics (e.g., high transsphincteric or low anterior), urgency of the procedure (elective or emergency), preoperative imaging (MRI or EUS), and adverse events such as seton loss or abscess recurrence.

Patient-reported outcomes were collected using a structured questionnaire administered either in person or via telephone. Patients were asked to rate each outcome as occurring “frequently,” “occasionally,” or “never.”

The assessed subjective variables included: Pain, Sitting discomfort, Discharge, Impaired hygiene, Foul odor, Fecal incontinence, Social impact, Sexual impact.

Fecal incontinence was assessed by asking patients to categorize their experience as “never,” “mild” (occasional staining or soiling), or “moderate” (frequent leakage or partial loss of control). While no validated scoring system was used, this simplified three-tier structure aligns with the lower descriptors of the Jorge–Wexner scale [6]. We acknowledge that mild incontinence may be difficult to distinguish from persistent fistula discharge, particularly in patients without prior baseline incontinence. However, all patients were followed by both surgical staff and specialized wound care nurses, and those reporting discomfort or hygiene-related concerns received closer clinical attention. This follow-up structure contributed to the consistency of patient reporting and mitigated the risk of systematic misclassification.

While the questionnaire was not based on previously validated instruments, it was applied consistently across all participants and time points, serving as the basis for both intraindividual and between-group comparisons. This method allowed for standardized collection of subjective patient experiences related to different seton characteristics.

#### Statistical analysis

McNemar’s test was used for intraindividual comparisons in the paired group. Chi-square or Fisher’s exact test was used for between-group comparisons. Statistical significance was set at p < 0.05. Analyses were conducted using IBM SPSS Statistics v29.0.

A planned multivariate logistic regression was not included in the final analysis due to sample size limitations.

#### Ethical approval and consent

The study was approved by the Ethics and Clinical Research Committee of University Hospital Infanta Leonor. All participants provided written informed consent.

## Results

A total of 33 patients underwent 53 seton placements. The mean age was 55.1 years (range 32–76), and 60.6% were male. Comorbidities included obesity in 42.4%, hypertension in 21.2%, and diabetes mellitus in 18.2%. A history of smoking was present in 66.7%, and Crohn’s disease in 9.1%. Most procedures were elective (72.7%), and 81.8% involved high transsphincteric fistulas. Preoperative imaging was performed in 63.6% of cases. A total of 27 O-shaped and 26 V-shaped setons were placed. Thin setons (1.5 mm) accounted for 73.6%, and thick ones (2.5 mm) for 26.4%. Laxity was ≤ 2 cm in 56.6% and > 2 cm in 43.4%. Full baseline characteristics are summarized in Table 1. The distribution of ASA physical status among the seton procedures is shown in Table 2.

**Table 1:** Baseline characteristics of the study population (n = 33 patients, 53 setons)

Variable	Value
Total patients	33
Total setons placed	53
Age, mean (range)	55.1 years (32–76)
Male sex	20 (60.6%)
Obesity (BMI > 30)	14 (42.4%)
Hypertension	7 (21.2%)
Diabetes mellitus	6 (18.2%)
Current or past smoker	22 (66.7%)
History of Crohn’s disease	2 (6.1%)
Elective procedure	24 (72.7%)
Emergency procedure	9 (27.3%)
High transsphincteric fistula	27 (81.8%)
Low fistula	6 (18.2%)
Preoperative imaging (MRI/EUS)	21 (63.6%)
O-shaped setons placed	27 (50.9%)
V-shaped setons placed	26 (49.1%)
Thin setons (1.5 mm)	39 (73.6%)
Thick setons (2.5 mm)	14 (26.4%)
Laxity ≤ 2 cm	30 (56.6%)
Laxity > 2 cm	23 (43.4%)

**Table 2:** ASA classification of the study population

ASA Classification	n (%)
I	15 (28.3%)
II	28 (52.8%)
III	10 (18.9%)

### Intraindividual comparison (paired group, n = 20)

Among patients who received both seton configurations, the O-shaped seton was associated with significantly less sitting discomfort compared to the V-shaped configuration (50.0% vs. 80.0%; p = 0.021). No other statistically significant differences were observed, with frequencies being similar across most outcome variables. Full results are shown in Table 3.

**Table 3:** Intraindividual comparison of O- and V-shaped setons (n = 20 pairs) *McNemar’s test used for all comparisons*

Variable	O-shaped (n = 20)	V-shaped (n = 20)	p-value
Sitting discomfort	10 (50.0%)	16 (80.0%)	0.021 *
Pain	4 (20.0%)	4 (20.0%)	1.000
Discharge	10 (50.0%)	11 (55.0%)	0.219
Impaired hygiene	5 (25.0%)	6 (30.0%)	1.000
Bad odor	3 (15.0%)	3 (15.0%)	1.000
Social impact	1 (5.0%)	1 (5.0%)	1.000
Sexual impact	4 (20.0%)	4 (20.0%)	1.000
Incontinence	3 (15.0%)	3 (15.0%)	1.000
Seton loss	1 (5.0%)	2 (10.0%)	1.000
Abscess recurrence	2 (10.0%)	2 (10.0%)	1.000

Outpatient seton replacement was successful in all cases, without complications or the need for surgical reintervention

### Unpaired group analysis (n = 13)

In the unpaired group, three separate subgroup analyses were conducted according to configuration, laxity, and thickness. No statistically significant differences were found in configuration- or thickness-based comparisons. However, laxity > 2 cm was associated with significantly higher incontinence (66.7% vs. 10.0%; p = 0.041). Detailed results are presented in Table 4, 5 and 6. Fisher’s exact test was used for all comparisons in the unpaired group.

**Table 4:** Comparison by configuration (unpaired group, n = 13)

Variable	O-shaped (n = 7)	V-shaped (n = 6)	p-value
Sitting discomfort	4 (57.1%)	3 (50.0%)	1.000
Pain	3 (42.9%)	2 (33.3%)	1.000
Discharge	6 (85.7%)	3 (50.0%)	0.317
Impaired hygiene	2 (28.6%)	2 (33.3%)	1.000
Bad odor	2 (28.6%)	1 (16.7%)	1.000
Social impact	1 (14.3%)	2 (33.3%)	0.553
Sexual impact	2 (33.3%)	2 (33.3%)	0.553
Incontinence	1 (14.3%)	1 (16.7%)	1.000
Seton loss	0 (0.0%)	1 (16.7%)	0.462
Abscess recurrence	1 (14.3%)	1 (16.7%)	1.000

**Table 5:** Comparison by laxity (unpaired group, n = 13)

Variable	Laxity > 2 cm (n = 3)	Laxity ≤ 2 cm (n = 10)	p-value
Sitting discomfort	2 (66.7%)	5 (50.0%)	1.000
Pain	2 (66.7%)	3 (30.0%)	0.517
Discharge	3 (100.0%)	6 (60.0%)	0.462
Impaired hygiene	2 (66.7%)	2 (20.0%)	0.205
Bad odor	2 (66.7%)	1 (10.0%)	0.111
Social impact	1 (33.3%)	2 (20.0%)	1.000
Sexual impact	2 (66.7%)	1 (10.0%)	0.111
Incontinence	2 (66.7%)	1 (10.0%)	0.041 *
Seton loss	0 (0.0%)	1 (10.0%)	1.000
Abscess recurrence	1 (33.3%)	1 (10.0%)	0.417

**Table 6:** Comparison by seton thickness (unpaired group, n = 13)

Variable	Thin (n = 8)	Thick (n = 5)	p-value
Sitting discomfort	4 (50.0%)	4 (80.0%)	0.305
Pain	2 (25.0%)	2 (40.0%)	0.596
Discharge	4 (50.0%)	3 (60.0%)	1.000
Impaired hygiene	3 (37.5%)	1 (20.0%)	0.590
Bad odor	2 (25.0%)	1 (20.0%)	1.000
Social impact	2 (25.0%)	1 (20.0%)	1.000
Sexual impact	2 (25.0%)	1 (20.0%)	1.000
Incontinence	1 (12.5%)	1 (20.0%)	1.000
Seton loss	1 (12.5%)	0 (0.0%)	1.000
Abscess recurrence	1 (12.5%)	1 (20.0%)	1.000

### Combined analysis of the full seton cohort (n = 53 cases)

In the full cohort of 53 seton placements, increased laxity was significantly associated with higher rates of discharge (86.4% vs. 51.6%; p = 0.008), sexual impact (42.1% vs. 10.3%; p = 0.016), and incontinence (31.8% vs. 12.9%; p = 0.041). A trend toward more pain with thick setons was observed (42.9% vs. 17.9%; p = 0.080), and impaired hygiene were more frequent with thin setons (30.8% vs. 7.1%; p = 0.075). Full results are summarized in Tables 7, 8 and 9. Comparisons in the combined analysis were performed using Fisher’s exact test or Chi-square test as appropriate. For greater precision, 95% confidence intervals were calculated for key outcomes and are presented in Table 10.

**Table 7:** Combined analysis by configuration (n = 53)

Variable	O-shaped (n = 27)	V-shaped (n = 26)	p-value
Pain	7 (25.9%)	7 (26.9%)	1.000
Sitting discomfort	15 (57.7%)	19 (70.4%)	0.354
Discharge	18 (66.7%)	16 (61.5%)	0.781
Impaired hygiene	7 (25.9%)	7 (26.9%)	1.000
Bad odor	4 (14.8%)	5 (19.2%)	0.727
Incontinence	3 (11.1%)	2 (7.7%)	1.000
Social impact	3 (11.1%)	4 (15.4%)	0.701
Sexual impact	6 (22.2%)	7 (26.9%)	0.671
Abscess recurrence	3 (11.1%)	4 (15.4%)	0.691
Seton loss	3 (11.1%)	2 (7.7%)	1.000

**Table 8:** Combined analysis by laxity (n = 53)

Variable	Laxity > 2 cm (n = 22)	Laxity ≤ 2 cm (n = 31)	p-value
Discharge	19 (86.4%)	16 (51.6%)	0.008 *
Pain	8 (36.4%)	5 (16.1%)	0.087
Sitting discomfort	14 (63.6%)	17 (54.8%)	0.520
Incontinence	7 (31.8%)	4 (12.9%)	0.041*
Impaired hygiene	5 (22.7%)	5 (16.1%)	0.719
Bad odor	3 (13.6%)	4 (12.9%)	1.000
Social impact	3 (13.6%)	2 (6.5%)	0.632
Sexual impact	8 (42.1%)	3 (10.3%)	0.016 *
Abscess recurrence	3 (13.6%)	4 (12.9%)	1.000
Seton loss	2 (9.1%)	3 (9.7%)	1.000

**Table 9:** Combined analysis by thickness (n = 53)

Variable	Thick (n = 14)	Thin (n = 39)	p-value
Discharge	9 (64.3%)	26 (66.7%)	1.000
Pain	6 (42.9%)	7 (17.9%)	0.080*
Sitting discomfort	9 (64.3%)	22 (56.4%)	0.749
Incontinence	1 (7.1%)	3 (7.7%)	1.000
Impaired hygiene	1 (7.1%)	12 (30.8%)	0.075*
Bad odor	2 (14.3%)	5 (12.8%)	1.000
Social impact	2 (14.3%)	3 (7.7%)	0.593
Sexual impact	3 (21.4%)	8 (20.5%)	1.000
Abscess recurrence	2 (14.3%)	5 (12.8%)	1.000
Seton loss	2 (14.3%)	3 (7.7%)	0.598

**Table 10:** Key patient-reported outcomes by seton characteristics, with 95% confidence intervals

Variable	Group	Events/Total	Frequency (%)	95% CI
Sitting discomfort	O-shaped seton	10/27	37.0%	21.5–55.8%
Sitting discomfort	V-shaped seton	14/26	53.8%	35.5–71.2%
Discharge	Laxity > 2 cm	19/22	86.4%	66.7–95.3%
Discharge	Laxity ≤ 2 cm	16/31	51.6%	34.8–68.0%
Sexual impact	Laxity > 2 cm	8/19	42.1%	23.1–63.7%
Sexual impact	Laxity ≤ 2 cm	3/29	10.3%	3.6–26.4%
Incontinence	Laxity > 2 cm	7/22	31.8%	16.4–52.7%
Incontinence	Laxity ≤ 2 cm	4/31	12.9%	5.1–28.9%
Pain	Thick seton	6/14	42.9%	21.7–66.5%
Pain	Thin seton	18/39	46.2%	31.6–61.4%
Impaired hygiene	Thick seton	1/14	7.1%	1.3–31.5%
Impaired hygiene	Thin seton	12/39	30.8%	18.1–47.1%

## Discussion

Our results demonstrate that specific seton characteristics—particularly configuration and laxity—significantly influence patient-reported comfort and clinical outcomes in the management of chronic perianal fistula. In the intraindividual analysis, the O-shaped seton configuration clearly reduced sitting discomfort compared to the V-shaped configuration (50.0% vs. 80.0%; p = 0.021). This finding is likely related to the mechanical behavior of the different designs: the closed loop of the O-shaped seton achieves a more uniform pressure distribution, whereas the free ends of the V-shaped configuration may cause localized irritation and increased pressure points on the perianal skin.

Excessive laxity (> 2 cm) was also significantly associated with increased discharge (86.4% vs. 51.6%; p = 0.008), fecal incontinence (66.7% vs. 10.0%; p = 0.041), and sexual impact (42.1% vs. 10.3%; p = 0.016). These results underline the importance of controlling seton tension during placement. Excessively lax setons can impair effective drainage and increase friction against surrounding skin, leading to greater discomfort and functional issues.

Regarding seton thickness, our findings revealed clinically suggestive trends that did not reach statistical significance. Thicker setons (2.5 mm) were associated with increased pain (42.9% vs. 17.9%; p = 0.080), while thinner setons (1.5 mm) showed a potential association with impaired hygiene (30.8% vs. 7.1%; p = 0.075). Although exploratory, these observations may help guide personalized seton selection based on patient tolerance and clinical presentation.

To our knowledge, this is the first study to systematically evaluate these specific technical aspects of seton placement using a prospective, comparative, and intraindividual design. Previous literature has primarily focused on functional classifications (e.g., cutting versus loose setons), overlooking more nuanced yet clinically meaningful technical choices [4, 5]. Our findings provide novel evidence that seemingly minor modifications in these parameters can significantly impact patient comfort and quality of life, guiding more informed surgical decision-making.

While these findings support the clinical relevance of seton selection, the study has certain limitations. The questionnaire was administered either in person or by telephone, which may have affected the accurate identification of subtle symptoms such as soiling versus fistula discharge. Nevertheless, all patients were clinically monitored during follow-up, and those reporting ambiguous symptoms underwent further assessment by surgical staff or wound care nurses. This ongoing oversight helped reduce potential misclassification bias and supported a more consistent collection of subjective patient experiences related to different seton characteristics.

The relatively small sample size reduced statistical power, particularly in subgroup analyses, and precluded the use of a multivariate logistic regression model due to separation of outcome variables. Some endpoints—such as pain and impaired hygiene—may have been based on limited patient representation, so the absence of statistically significant results for these outcomes should be interpreted with caution. We also acknowledge that certain subgroup comparisons involved very small absolute numbers, limiting their clinical reliability (e.g., 2 vs. 1 case in some incontinence analyses). These findings are exploratory in nature and not intended to support definitive conclusions. Nonetheless, the consistency of trends across both the paired and the full cohort—including associations between excessive laxity and outcomes such as discharge, sexual impact, and incontinence—supports their hypothesis-generating value and underscores the need for confirmatory studies with formal sample size calculations.

The exclusive use of silicone vessel loops was a deliberate methodological choice to minimize variability and reduce potential confounding factors. While this approach prevented the evaluation of material-related differences, it allowed for a more controlled analysis of the impact of seton configuration, thickness, and laxity.

The subjective nature of patient-reported outcomes inevitably introduces individual variability. However, the consistent use of structured questionnaires and the intraindividual design of part of the study helped to mitigate this limitation and enhance internal validity.

Clinically, our findings support recommending O-shaped seton configurations and careful control of laxity (≤ 2 cm), particularly in patients with existing discomfort or concerns about perianal irritation. These technical considerations may directly improve patient comfort and treatment acceptance.

Future multicentric studies with larger sample sizes are needed to validate our findings and explore other relevant variables, such as seton materials, interactions with biological therapies, and detailed assessments of patient-reported outcomes. In the meantime, we recommend applying the technical considerations described here to optimize the clinical management of patients requiring prolonged seton drainage for chronic perianal fistula.

## Conclusions

Seton configuration and laxity appear to influence patient-reported comfort. O-shaped setons were associated with less sitting discomfort, while excessive laxity (> 2 cm) correlated with more discharge, sexual impact, and incontinence. Thickness showed less consistent effects, with trends toward more pain in thicker setons and more hygiene issues in thinner ones.

Tailoring seton placement to minimize laxity and consider configuration may help improve tolerance. Further studies are needed to validate these findings.

## Supplementary Information

Below is the link to the electronic supplementary material.

Supplementary file 1 (DOCX 17.1 KB)

## Data Availability

No datasets were generated or analysed during the current study.

## Declarations

### Data Sharing Statement

The data that support the findings of this study are available from the corresponding author upon reasonable request. Access to de-identified patient data will be granted to researchers with a methodologically sound proposal, following institutional and ethical guidelines. Requests should be directed to analeonbre@gmail.com.
