# Supplementary material for: Influence of seton configuration, thickness, and laxity on patient comfort in chronic perianal fistula: a prospective comparative study
Source: Int J Colorectal Dis. 2025 Aug 25;40(1):187. doi: 10.1007/s00384-025-04985-9 (PMC12378777; doi:10.1007/s00384-025-04985-9)
Supplement: Supplementary file 1 — (DOCX 17.1 KB) [file 384_2025_4985_MOESM1_ESM.docx]

**APPENDIX 1 – PATIENT QUESTIONNAIRE (translated from Spanish)**

NHC:
CODE:
DATE OF INITIAL SETON PLACEMENT:
DATE OF STUDY INCLUSION (IC SIGNATURE):

PERSONAL DATA

- Age:
- Sex (0- Male; 1- Female):
- Diabetes Mellitus (DM) (0- No; 1- Yes):
- Hypertension (HTN) (0- No; 1- Yes):
- Smoking (0- No; 1- Yes; 2- Former smoker):
- Obesity BMI > 30 (0- No; 1- Yes):
- BMI:
- ASA Score (1; 2; 3; 4; 5):
- Associated diseases:
- Inflammatory Bowel Disease (IBD) (0- No; 1- Yes):
  - Systemic treatment for IBD (0- No; 1- Yes):
  - Type of systemic treatment (0- Corticosteroids; 1- Biologics; 2- Others):
- Fistula origin: (0- Cryptoglandular; 1- Ulcer (CD); 2- Fissure; 3- Iatrogenic):
- Number of fistulas:
- Number of external openings (OFEs):
- Number of internal openings (OFIs):
- First surgical intervention (0- No; 1- Yes):
- Number of previous interventions:
  - Type of previous intervention (0- Primary closure; 1- Flap; 2- LIFT; 3- Plug; 4- Laser; 5- Cell therapy; 6- PRP):
- Recurrence after seton removal and repair (0- No; 1- Yes):
  - Number of recurrences:
- Fistula type: (1- Submucosal; 2- Intersphincteric; 3- Low transsphincteric; 4- High transsphincteric; 5- Extrasphincteric):
- Rectovaginal fistula (0- No; 1- Yes):
- Surgery type for seton placement (0- Urgent; 1- Elective):
- Additional procedures during surgery (0- None; 1- Anal abscess drainage; 2- Cavity curettage; 3- Enucleation of extrasphincteric tract; 4- Simplification of multiple OFEs):
- Preoperative imaging performed (1- Yes; 2- No):
  - Which imaging test? (1- CT; 2- MRI; 3- Ultrasound):
- Anal incontinence (1- Yes; 2- No):
  - Type of incontinence:
- Seton placed by colorectal surgeon? (1- Yes; 2- No):
- Completed follow-up? (1- Yes; 2- No):
  - Reason for not completing follow-up? (1- Healed; 2- Patient refusal; 3- Death; 4- Moved to another area; 5- Met an exclusion criterion):

REVIEW 1 (3 MONTHS)

- Date:
- Type of seton: (0- O-shaped; 1- V-shaped):
- Seton thickness: (0- Thin; 1- Thick):
- Seton laxity (skin to closure distance): (0 < 2cm; 1 > 2cm):
- Number of fixation sutures (1- 1; 2- 2; 3- 3):
- Pain (0- No; 1- Yes):
- VAS (Visual Analog Scale):
- Discharge (0- No; 1- Yes):
- Fever (0- No; 1- Yes):
- Discomfort when sitting (0- Never; 1- Occasionally; 2- Frequently):
- Hygiene difficulty (0- Never; 1- Occasionally; 2- Frequently):
- Bad odor (0- Never; 1- Occasionally; 2- Frequently):
- Impact on social relationships (0- Never; 1- Occasionally; 2- Frequently):
- Impact on sexual relationships (0- Never; 1- Occasionally; 2- Frequently):
- Abscess recurrence (0- No; 1- Yes):
- Date of recurrence:
- Seton loss (0- No; 1- Yes):
  - Date of loss:

REVIEW 2 (3 MONTHS) (Change type of seton at 3-month review: If V, change to O and vice versa)

- Date:
- Type of seton: (0- O-shaped; 1- V-shaped):
- Seton thickness: (0- Thin; 1- Thick):
- Seton laxity (skin to closure distance): (0 < 2cm; 1 > 2cm):
- Number of fixation sutures (1- 1; 2- 2; 3- 3):
- Pain (0- No; 1- Yes):
- VAS (Visual Analog Scale):
- Discharge (0- No; 1- Yes):
- Fever (0- No; 1- Yes):
- Discomfort when sitting (0- Never; 1- Occasionally; 2- Frequently):
- Hygiene difficulty (0- Never; 1- Occasionally; 2- Frequently):
- Bad odor (0- Never; 1- Occasionally; 2- Frequently):
- Impact on social relationships (0- Never; 1- Occasionally; 2- Frequently):
- Impact on sexual relationships (0- Never; 1- Occasionally; 2- Frequently):
- Abscess recurrence (0- No; 1- Yes):
- Date of recurrence:
- Seton loss (0- No; 1- Yes):
  - Date of loss:
